# Modified Negative Staining of Heine for Fast and Inexpensive Screening of *Cryptosporidium*, *Cyclospora*, and *Cystoisospora* spp.

**DOI:** 10.1155/2014/165424

**Published:** 2014-10-20

**Authors:** Vinay Khanna, Kriti Tilak, Archi Ghosh, Chiranjay Mukhopadhyay

**Affiliations:** Department of Microbiology, Kasturba Medical College, Manipal University, 576104 Karnataka, India

## Abstract

Negative staining technique of Heine is an easy, inexpensive, and rapid way of screening for coccidian parasites of the intestinal tract. But its use as a routine technique for screening of *Cryptosporidium*, *Cyclospora*, and *Cystoisospora* is restricted due to its sensitivity being lower than the gold standard method of modified Ziehl-Neelsen staining. This paper emphasises the modification of original Heine staining technique which has been attempted in order to increase the sensitivity and detection of oocysts of *Cryptosporidium*, *Cyclospora*, and *Cystoisospora*. Modified Heine staining technique using malachite green is a practical, safe, and sensitive method of detecting oocysts in stool specimens. While the modified Ziehl-Neelsen staining technique is still considered the gold standard for the detection of *Cryptosporidium* spp., modified negative staining technique of Heine using malachite green stain should be considered as the screening technique of first choice.

## 1. Introduction

*Cryptosporidium *spp. and* Cystoisospora belli* are intracellular, extracytoplasmic coccidian parasites of the intestinal tract and have been recognized as a pathogen in humans since 20th century [1]. It is a well-acknowledged fact that* Cryptosporidium* and* Cystoisospora*, which were earlier assumed to be the causal agents of acute diarrhoea merely in animals, have now emerged as one of the leading causes of prolonged life threatening diarrhoea in immunocompromised patients particularly in patients with AIDS. Oocysts of* Cryptosporidium* and* Cyclospora* are spherical in shape and 4–6 *μ*m and 8–10 *μ*m in diameter, respectively. In contrast, oocysts of* Cystoisospora belli* are generally ovoid to ellipsoid in shape and 10–40 *μ*m in length by 10–30 *μ*m in width and may contain specialized structures, such as polar caps, micropyles, and residual and crystalline bodies.* Cryptosporidium* oocysts exhibit acid-fast staining and must be differentiated from partially acid-fast organisms including* Cyclospora cayetanensis *[2]. Diagnosis is made by examination of stool samples for oocysts by direct microscopy. Microscopic examination of stool specimens using various techniques (e.g., acid-fast staining and direct fluorescent antibody (DFA)) is the most frequently employed practice. Concentration of oocysts by sedimentation or flotation may improve detection by microscopic examination [3].

The negative staining technique of Heine is a simple, inexpensive, and efficient way of screening for* Cryptosporidium parvum* and* Cystoisospora belli* [4]. Enzyme immunoassays (for detection of* Cryptosporidium* specific antigens) and molecular methods (e.g., polymerase chain reaction (PCR)) are increasingly used in reference diagnostic laboratories. PCR can be used to identify the organisms at the species level [5]. More recently, Varma et al. [6] developed a real-time PCR assay for the detection of* Cyclospora* oocysts. However, the detection of* Cryptosporidium* and* Cyclospora *in India is restricted to major research laboratories and does not form a part of protocols for routine investigations in most clinical laboratories; therefore, health care providers should precisely request testing for these parasites.

## 2. Objectives

The objectives of this paper are as follows:

1. to compare sensitivity of negative staining techniques using various stains such as malachite green, crystal violet, and methylene blue for rapid detection of oocysts of* Cryptosporidium*,* Cyclospora*, and* Cystoisospora*;
2. to compare the sensitivity of negative staining of Heine with modified negative staining method.

## 3. Material and Methods

We screened one hundred fresh stool samples, not conserved in formalin, over a study period of one year, obtained from immunocompromised patients with history of diarrhoea in tertiary care hospital, by negative staining of Heine. Heine technique of negative staining uses carbol fuchsin for staining the stool smear. Since we found the sensitivity of Heine technique lower than that of gold standard method of modified Ziehl-Neelsen staining, we attempted a modification in the original Heine staining procedure, in order to increase the detection rate of oocysts. The original Heine staining procedure was modified by staining stool smears separately using malachite green, methylene blue, and crystal violet, in place of carbol fuchsin. Concentrated stool smears confirmed positive for oocysts by modified acid-fast staining were considered for modified negative staining technique. Thin smears were prepared by mixing equal amount of undiluted carbol fuchsin, malachite green, methylene blue, and crystal violet solution with concentrated faecal sample on a glass slide. Smears were allowed to air-dry and examined using 10x eyepiece and in oil-immersion objective. Blinded smears stained with malachite green, methylene blue, crystal violet, and carbol fuchsin were given to ten microbiologists for further identification of oocysts.

## 4. Results

Amongst hundred stool sample smears stained with modified acid-fast technique, eight were found to be positive for oocysts of* Cryptosporidium*, six were positive for oocysts of* Cystoisospora belli*, and two were positive for oocysts of* Cyclospora* spp. Smears were prepared from all positive stool samples and stained separately with malachite green, methylene blue, crystal violet, and carbol fuchsin. On microscopic examination of negatively stained smears all oocysts appeared as unstained, strongly refractile, and round to oval structures against dark coloured background. Internal structures were slightly visible as darker specks inside the oocyst.

**Figure 1:**
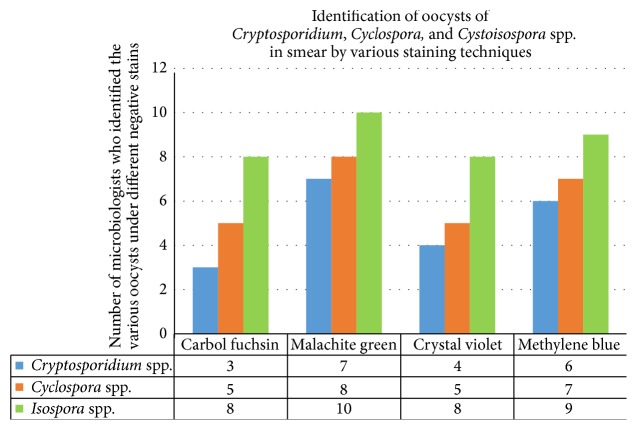
Identification of oocysts of* Cryptosporidium*,* Cyclospora*, and* Cystoisospora *spp. in smear stained with various staining techniques.

Ten microbiologists were requested to identify oocysts of parasites in all the smears. In the smear stained with carbol fuchsin,* Cryptosporidium*,* Cyclospora*, and* Cystoisospora *cysts were visible to 3, 5, and 8 microbiologists, respectively. Considerably higher number (7, 8, and 10, resp.) of microbiologists could identify the cysts in the smear stained with malachite green. In the smear stained with crystal violet, 4, 5, and 8 microbiologists could identify* Cryptosporidium*,* Cyclospora*, and* Cystoisospora* cysts, respectively, while 6, 7, and 9 could identify them in the smear stained with methylene blue (Figure 1).

**Figure 2:**
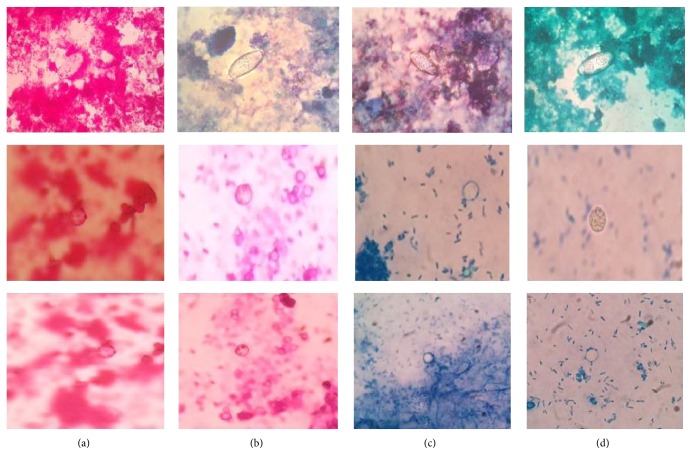
Various negatively stained smears showing* Cystoisospora *spp.,* Cyclospora *spp., and* Cryptosporidium *spp. (a) Carbol fuchsin stained smear showing* Cystoisospora* spp.,* Cyclospora* spp., and* Cryptosporidium* spp. (b) Crystal violet stained smear showing* Cystoisospora* spp.,* Cyclospora* spp., and* Cryptosporidium* spp. (c) Methylene blue stained smear showing* Cystoisospora* spp.,* Cyclospora* spp., and* Cryptosporidium* spp. (d) Malachite green stained smear showing* Cystoisospora* spp.,* Cyclospora* spp., and* Cryptosporidium* spp.

By staining with carbol fuchsin, methylene blue, malachite green, and crystal violet, oocysts could be seen as refractile structures. The stain particles of methylene blue decreased the refractility of the structures. Malachite green and crystal violet improved the refractility of the structures the most.* Cystoisospora* oocysts could be distinctly seen as unstained structures against green and violet background, respectively. Internal morphology (sporozoites) was visible in some* Cystoisospora* cysts under oil immersion (100x magnification) lens. With the malachite green stain, yeasts were clearly differentiated from oocysts as they took up the stain. Majority of the microbiologists found it easier to identify the structures in staining technique which used malachite green compared to the other three staining techniques done for identification of parasitic cysts, because malachite green provided maximum contrast between the oocysts and the background (Figure 2).

## 5. Discussion

A variety of methods have been developed for the detection of* Cryptosporidium *spp.,* Cystoisospora*, and* Cyclospora* which include microscopic, immunological, and molecular techniques. Immunological and molecular techniques are more time-consuming, complex, and expensive, making them less beneficial methods for screening, especially in resource-poor settings. However, they have usually better sensitivities and specificities. Effective diagnosis of infections caused by these coccidian parasites requires diagnostic tools to be time-saving, cost-effective, accurate, and sensitive. As microscopy is a speedy, economical, and reliable diagnostic tool, it can be used for screening in primary health care settings as well. Microscopic detection is based on finding the environmentally and chemically resistant oocysts in the stool samples. It provides the advantage of direct visual confirmation of the presence of* Cryptosporidium, Cystoisospora*, and* Cyclospora* oocysts. This paper appreciates the negative staining technique of Heine as an easy, inexpensive, and efficient way of screening for* Cryptosporidium *spp. A modification in the original Heine staining technique has been attempted in order to increase the sensitivity of the original technique. Modified Heine staining technique, using malachite green, is a practical, safe, and sensitive method of detecting oocysts in stool specimens. Because of the ease with which oocysts can be differentiated from yeasts, less experienced microbiologists can still accurately diagnose the oocysts. The sensitivity of this technique can be further increased by using phase-contrast microscopy or examination at 400x magnification. To further improve the sensitivity of the negative staining technique, we recommend adding a drop of oil on the slide and covering it with a coverslip. This enables the microbiologist to observe the preparation using 10x eyepieces and a “dry” objective of 40x magnification.

Some of the shortcomings of the technique are as follows. (1) Thickness of the smear adversely affects the visibility of the cysts. Thin smears are favourable for better identification of the cysts. (2) Presence of debris in the stool sample reduces the probability of identification of cysts in the smear. (3) In order to achieve better results, stain has to be mature and needs to be filtered to reduce the amount of stain particles and hence increase the clarity.

## 6. Conclusion

In our modified negative staining method, since malachite green dye does not penetrate the cell, the results yield a clear structure against a dark background. Since it does not necessitate heat fixation or other treatments such as decolourization with solvents or staining cells with cationic dyes, consequently, cells revealed by negative staining remain as close to their native shapes and sizes as possible. Thus, the main advantage and use of this technique are that it allows examination of the normal morphology of microorganisms. Another advantage is that the technique involves the use of only one stain, therefore making it economical. Further, this technique is immensely beneficial in screening the stool samples for the parasitic cysts rapidly and effortlessly. Greater contrast between the cysts and the background makes the cysts easy to be identified. This will be particularly valuable for unskilled students doing microscopic examinations for stool parasites.

While the modified Ziehl-Neelsen staining technique is still considered the gold standard for the detection of* Cryptosporidium *spp.,* Cystoisospora*, and* Cyclospora*, modified negative staining technique of Heine using malachite green stain should be considered as the screening technique of first choice.
